# Visual field defects and changes in central retinal artery occlusion

**DOI:** 10.1371/journal.pone.0209118

**Published:** 2019-01-03

**Authors:** Hyeong Min Kim, Young Joo Park, Kyu Hyung Park, Se Joon Woo

**Affiliations:** Department of Ophthalmology, Seoul National University College of Medicine, Seoul National University Bundang Hospital, Seongnam, Republic of Korea; Massachusetts Eye & Ear Infirmary, Harvard Medical School, UNITED STATES

## Abstract

**Objectives:**

To investigate the characteristics and temporal changes in visual field defects (VFDs) in eyes with acute central retinal artery occlusion (CRAO).

**Design:**

Retrospective, observational case series

**Methods:**

A total of 119 patients diagnosed with acute non-arteritic CRAO through examination with Goldmann perimetry were included among the patients who visited Seoul National University Bundang Hospital between January 2009 and December 2016. They were treated with either conservative treatments or intra-arterial thrombolysis (IAT). The baseline features and temporal changes of visual field examination results and the association with clinical parameters including visual acuity, optical coherence tomography (OCT) findings, and the CRAO stages.

**Results:**

All of the 119 patients showed visual field defect and suffered unilateral acute CRAO. We observed five characteristic VFDs: peripheral constriction only (8%), paracentral scotoma (3%), central and cecocentral scotoma (19%), temporal island (59%), and no visual field (10%). Severe VFDs were associated with severe CRAO stages, poor baseline BCVA, delayed retinal arterial perfusion, and severe retinal morphologic changes on OCT. We found improvements in the visual field in 39% of all cases during the follow-up periods. Mild CRAO stages, good baseline BCVA, mild retinal morphologic changes, and mild initial VFDs were significantly associated with visual field improvement.

**Conclusions:**

The five characteristic types of VFDs and their improvement in eyes with CRAO are associated with baseline features related to the severity of retinal ischemia. We suggest that the underlying mechanisms of VFDs involve the balance between the retinal arterial perfusion and the ischemic vulnerability of each retinal area.

## Introduction

Central retinal artery occlusion (CRAO) is an ischemic retinopathy with abrupt and devastating visual loss caused by the acute diminution of arterial perfusion of the inner retinal layers. [1–3] Over 90% of patients with CRAO experience permanent loss of vision with a final visual acuity of counting fingers or less. [1–6] CRAO occurs with a spectrum of severity and can be classified according to the degree of retinal ischemia and features on ophthalmic examinations. Schmidt and colleagues categorized CRAO as incomplete, subtotal, or total CRAO. [7] Incomplete CRAO is characterized by diminished VA, slight retinal edema with indefinite cherry-red spots, and mildly delayed retinal arterial perfusion on fluorescein angiography (FA). Subtotal CRAO is identified as severe reduction in VA, distinct retinal edema with cherry-red spots, and severely delayed retinal arterial perfusion. Total CRAO is characterized by severe retinal ischemia and massive retinal edema in the macula often accompanied by choroidal perfusion delay and no light perception. [7] Our previous studies have revealed that the stage of CRAO is significantly related to the degree of vision loss, extent of retinal edema detected by optical coherence tomography (OCT), delay in arterial blood flow, and treatment outcomes. [8, 9]

Visual field defects (VFDs) as an outcome of CRAO is relatively less studied compared to visual acuity. Hayreh and Zimmerman reported the classification of central and peripheral visual fields in 145 eyes with CRAO. [6] In the study, common types of VFDs were “no peripheral defect or constricted field,” “central scotoma,” and “temporal island.” Central and peripheral visual fields showed improvement in part of patients with non-arteritic CRAO. However, the association between the visual field and other clinical factors of CRAO has not yet been investigated. In addition, although the European Assessment Group for Lysis in the Eye (EAGLE) trial [10] reported no significant visual improvement by intra-arterial fibrinolysis, it is possible that early restoration of retinal arterial perfusion might be beneficial to the peripheral visual field. In this study, we focused on the features and the temporal changes in VFDs in the different stages of CRAO. In addition, we found that the features of the visual fields were correlated with other clinical characteristics such as visual acuity, retinal perfusion time, and OCT findings. We also suggested a mechanism underlying VFDs in eyes with various stages of CRAO.

## Materials and methods

### Patient selection and intervention performed

The institutional review board of Seoul National University Bundang Hospital approved this retrospective study, and the study adhered to the tenets of the Declaration of Helsinki.

**Fig 1:**
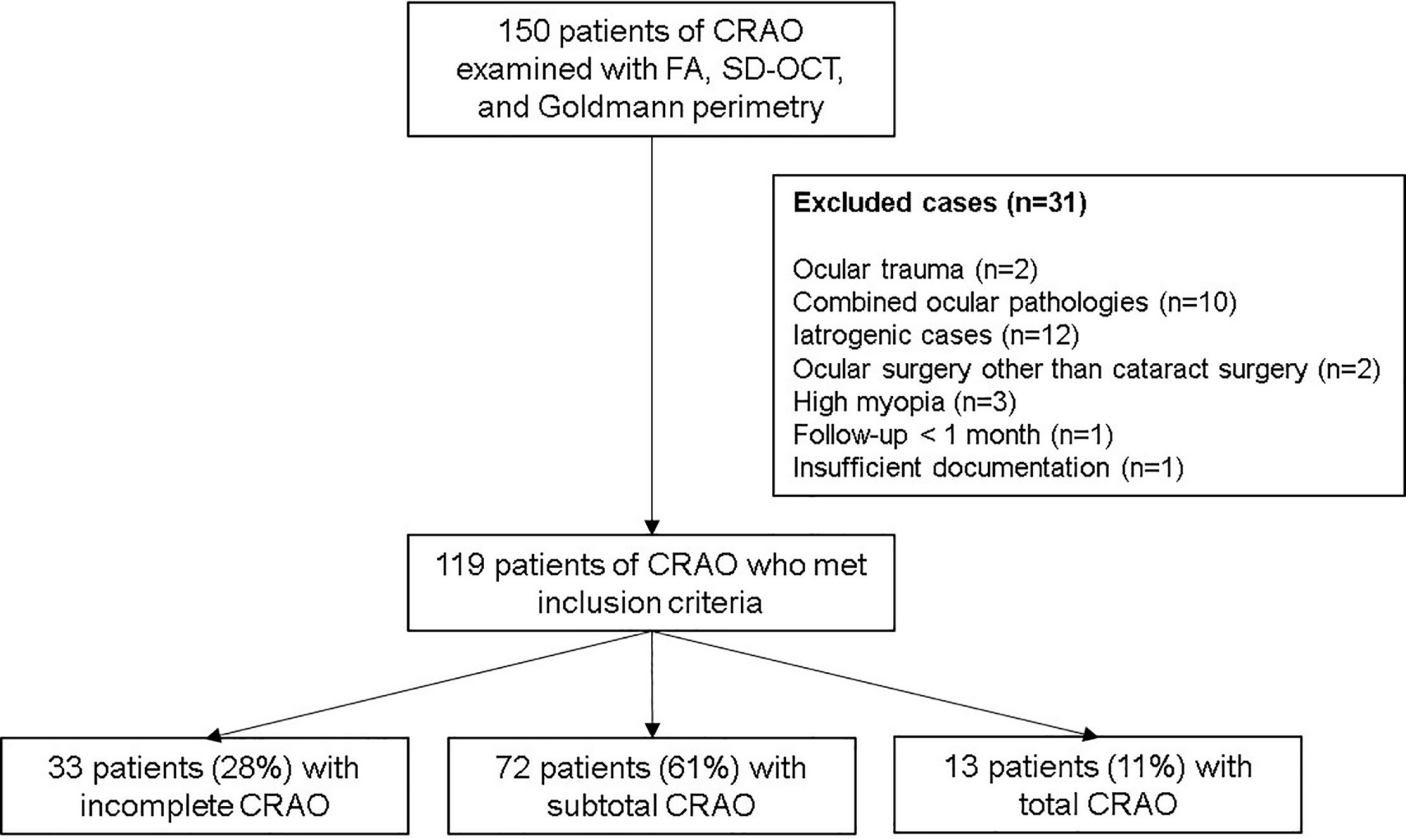
Flow diagram showing the selection process of the enrolled eyes with central retinal artery occlusion. CRAO = central retinal artery occlusion; FA = fluorescein angiography; SD-OCT = spectral-domain optical coherence tomography.

The present study reviewed 150 eyes from 150 patients diagnosed with acute non-arteritic CRAO who (1) had visited Seoul National University Bundang Hospital between January 2009 and December 2016, over an 8-year-period, for vision loss or a visual field defect occurring within 14 days of the initial visit and (2) had undergone FA (VX-10; Kowa OptiMed, Tokyo, Japan), spectral domain optical coherence tomography (SD-OCT, Spectralis OCT; Heidelberg Engineering Inc, Heidelberg, Germany), and Goldmann perimetry evaluation at the initial visit. The exclusion criteria were as follows: a history of ocular trauma (n = 2); combined ocular pathologies such as macular disease, severe nonproliferative or proliferative diabetic retinopathy, ocular ischemic syndrome and central retinal vein occlusion (n = 10); iatrogenic cases of CRAO (n = 12); ocular surgery other than cataract surgery (n = 2); high myopia (>6 diopters, n = 3); follow-up of less than 1 month (n = 1); and insufficient documentation of ocular evaluation (n = 1). In total, we included 119 eyes from 119 patients with acute non-arteritic CRAO in the analyses (Fig 1).

We treated patients with non-arteritic CRAO with either conservative treatments or intra-arterial thrombolysis (IAT), as described in our previous study. [8] We performed IAT for patients in whom visual improvement might be expected; these patients had the following characteristics: (1) delayed retinal arterial perfusion on FA (2) symptom duration less than 24 hours for subtotal or total CRAO patients, and less than 1 week for incomplete CRAO patients. The conservative treatments consisted of ocular massage (repeated manual compression for 10 to 15 seconds followed by sudden release), pressure-lowering agents (topical timolol 0.5%, oral acetazolamide 500 mg), or no treatment. We performed IAT together with cerebral angiography, in which the microcatheter (Excelsior SL-10; Stryker Neurovascular, Fremont, California, USA) was placed in the proximal segment of the ophthalmic artery, and up to 500,000 units of urokinase (Green Cross, Yongin, South Korea) were injected slowly by hand until visual improvement was noted.

### Ophthalmic examinations

All patients underwent complete ophthalmic evaluations including best-corrected visual acuities (BCVA), slit lamp biomicroscopy, indirect ophthalmoscopy, fundus photography, fluorescein angiography, SD-OCT imaging, and Goldmann perimetry. To quantify the degree of perfusion state, we measured the FA arm-to-retina time and arteriovenous passage time at pretreatment and post-treatment follow-up visits [11, 12]. FA was repeated until the complete retinal arterial reperfusion was observed. With the OCT images, central macular thickness (CMT) and the structural morphologic changes were measured in the same manner as our previous study [9]. Specifically, structural changes in OCT were classified to: inner retinal hyperreflectivity only, retinal thickening and loss of layer structure, and presence of retinal fluid.

To investigate the VFDs in patients with CRAO, we plotted the Goldmann perimetry for each visit. With reference to previous studies on visual fields [13, 14], we named five representative types of VFDs in order of severity: peripheral constriction only, paracentral scotoma, central and cecocentral scotoma, temporal island, and no visual field. Small visual field islands in superior or inferior region were included in the temporal island group, considering lack of accurate fixation in CRAO patients. We designated patients to the no visual field classification if they could not detect even the V-4e isopters of size and light intensity.

Visual field improvement was defined as the combination of (1) quantitative enlargement of the visual field at the time of the final perimetry, compared to that at the initial perimetry and (2) the patients’ subjective perception of visual field improvement. Both criteria should be fulfilled to be classified as ‘visual field improvement’. It was evaluated in only 72 patients who underwent at least two sequential visual field tests and the questionnaire which addresses subjective visual field improvement. For multiple follow-up visual field tests, the most improved (or the largest) state of visual field was chosen as the final perimetry for comparison. At least 6 months interval between the initial and the final visual field tests was set to observe the change sufficiently.

### Statistical analyses

We compared demographics and clinical data including visual fields, visual acuity, and OCT features according to the stages of CRAO. Data for continuous variables are expressed as the mean ± standard deviation. We converted Snellen VA measurements into logarithmic minimum angle of resolution (logMAR) values for statistical analysis. The numerical scores for profound low vision, namely, counting fingers or worse, were substituted for logMAR values, as suggested by Lange et al (finger count: 2.0; hand motion: 2.3; light perception: 2.6; no light perception: 2.9). [15] Frequency data were compared using Fisher exact test, and the continuous variables were compared using analysis of variance for parametric data and Kruskal-Wallis test and Mann-Whitney U test for nonparametric data, depending on normality determined by the Shapiro-Wilk test. We analyzed associations between CRAO stages and the incidence of visual field improvement using the Fisher’s exact test. To identify associative factors for the severity of visual field type and visual improvement, we performed additional analyses including the Mann-Whitney U test, independent t-test, and Fisher’s exact test, according to the variables. We performed multivariate analysis for visual field improvement using binary logistic regression analysis via 2 separate models considering multicollinearity and small population size. We performed all statistical analyses using SPSS software version 21.0 for Windows (SPSS, Inc, Chicago, Illinois, USA), and a *P* value less than .05 was considered to indicate a statistically significant difference.

## Results

### Clinical characteristics of patients

Of 150 non-arteritic CRAO patients evaluated over an 8-year period, 119 CRAO patients met the inclusion criteria. Also, all of the 119 patients showed visual field defect and suffered unilateral acute CRAO. The detailed demographics and clinical characteristics are summarized in Table 1. The mean age was 60.8±15.2 years and the male to female ratio was 73:46. The mean follow-up period was 13.7±16.1 months. Mean baseline and final BCVA were 2.22±0.44 and 1.98±0.71, and mean time from symptom onset to treatment was 32.2±50.4 hours. FA arm-to-retina time was 23.6±8.9 seconds. The patients were categorized according to the CRAO stage: incomplete CRAO, 33 (28%); subtotal CRAO, 73 (61%); and total CRAO, 13 (11%) (Fig 1). The initial and final BCVAs were significantly different among CRAO subgroups (*P* < 0.001, Table 1). There were significant trends of worsening VA, prolonged FA arm-to-retina time and worsening OCT features, according to the stages of CRAO(*P* < .001, *P* = .025, and *P* < .001, respectively, Table 1). In addition, the post-hoc analyses were done among CRAO subgroups in VA, FA arm-to-retina time and OCT features. For visual acuity, baseline and final BCVA were significantly different among each subgroups (all *P* < .001). However, prolonged FA arm-to-retina time was significantly different only between total and other subgroups (*P* = .021, *P* = .034) but no difference between incomplete and subtotal subgroups (*P* = .822). For OCT features, baseline central macular thickness differed among each subgroups (all *P* < .001), but the final CMT did not differ significantly between subtotal and total subgroup (*P* = .841), while incomplete and other subgroups differed (*P* = .003, *P* = .046).

**Table 1:** Demographics and clinical characteristics of CRAO patients and comparisons based on the disease stage.

Variables	All CRAO (N = 119)	Incomplete (N = 33)	Subtotal (N = 73)	Total (N = 13)	P^*^^†^^‡^ (Incomplete vs Subtotal vs Total)	Incomplete vs Subtotal	Incomplete vs Total	Subtotal vs Total
Mean age	60.8±15.2 (17, 88)	60.3±13.2 (32, 83)	62.6±13.7 (17, 88)	52.4±24.6 (18, 79)	.081†			
Male : Female	73 : 46	26 : 7	42 : 31	5 : 8	.023^*^			
Standard treatment : Intra-arterial thrombolysis	46 : 73	16 : 17	25 : 48	5 : 8	.378^*^			
Mean time from symptom onset to treatment (hour)	32.2±50.4 (1, 336)	51.6±60.2 (1, 168)	26.1±47.1 (1, 336)	15.4±18.2 (1, 72)	**.038**‡			
Mean follow-up period (month)	13.7±16.1 (1, 98)	17.4±16.2 (1, 57)	13.1±16.9 (1, 98)	7.9±8.1 (2, 26)	.117‡			
FA arm-to-retina time (sec)	23.6±8.9 (5, 55)	22.2±6.9 (7, 42)	23.3±8.9 (5, 47)	31.1±12.7 (11, 55)	**.025**†	.822	**,021**	**.034**
Visual acuity (logMAR)								
Mean baseline BCVA	2.22±0.44 (20/90, NLP)	1.83±0.53 (20/90, HM)	2.30±0.23 (20/1000, NLP)	2.78±0.23 (HM, NLP)	**< .001**‡	**< .001**	**< .001**	**< .001**
Mean final BCVA	1.98±0.71 (20/18, NLP)	1.25±0.78 (20/18, HM)	2.18±0.41 (20/300, HM)	2.71±0.29 (HM, NLP)	**< .001**‡	**< .001**	**< .001**	**< .001**
OCT features								
At baseline					< .001*			
Inner retinal hyperreflectivity only	45 (38%)	29 (88%)	16 (22%)	0				
Retinal thickening and loss of layer structure	56 (46%)	4 (12%)	48 (66%)	4 (31%)				
Retinal fluid	18 (15%)	0	9 (12%)	9 (69%)				
At the final examination								
Foveal photoreceptor defect	80 (67%)	8 (24%)	59 (81%)	13 (100%)	< .001*			
Mean baseline CMT (μm)	390.6±163.5 (205, 1176)	281.0±33.9 (205, 340)	409.2±146.0 (228, 925)	598.1±250.6 (383, 1176)	**< .001**‡	**< .001**	**< .001**	**< .001**
Mean final CMT (μm)	223.0±36.8 (121, 378)	233.6±24.9 (165, 286)	219.7±39.3 (151, 378)	212.3±45.5 (121, 287)	**.013**‡	**.003**	**.046**	.841
Baseline visual field features					< .001*			
Peripheral constriction only	10 (8%)	10 (30%)	0	0				
Paracentral scotoma	4 (3%)	4 (13%)	0	0				
Central/cecocentral scotoma	23 (19%)	10 (30%)	13 (18%)	0				
Temporal island	70 (59%)	9 (27%)	58 (80%)	3 (23%)				
No visual field	12 (10%)	0	2 (2%)	10 (77%)				
Visual field improvement^¶^	28/72 (39%)	18/28 (64%)	10/41 (24%)	0/3 (0)	< .001*			

*P* values in boldface indicate statistical significance.

* Fisher exact test

† One-way ANOVA for continuous parametric variables

‡ Kruskal-Wallis test and Mann-Whitney U test for continuous nonparametric variables

¶Visual field improvement was evaluated in only those underwent at least two sequential visual field tests (N = 72)

CRAO, central retinal artery occlusion; FA, fluorescein angiography; logMAR, logarithm of the minimal angle of resolution; HM, hand motion; NLP, no light perception; BCVA, best-corrected visual acuity; OCT, optical coherence tomography; CMT, central macular thickness

73 patients (61.3%) received intra-arterial thrombolysis while the others (46 patients, 38.7%) received conservative treatment. The amount of visual improvement at the final visit was significantly different among patients who underwent different treatments (conservative treatment vs IAT) in the incomplete CRAO group, reconfirming the findings of our prior study [8] (-0.18 ± 0.44 [ST group, n = 16] vs. -0.96 ± 0.66 logMAR [IAT group, n = 17], *P* = 0.001).

### Visual field defects (VFDs) in CRAO patients

**Fig 2:**
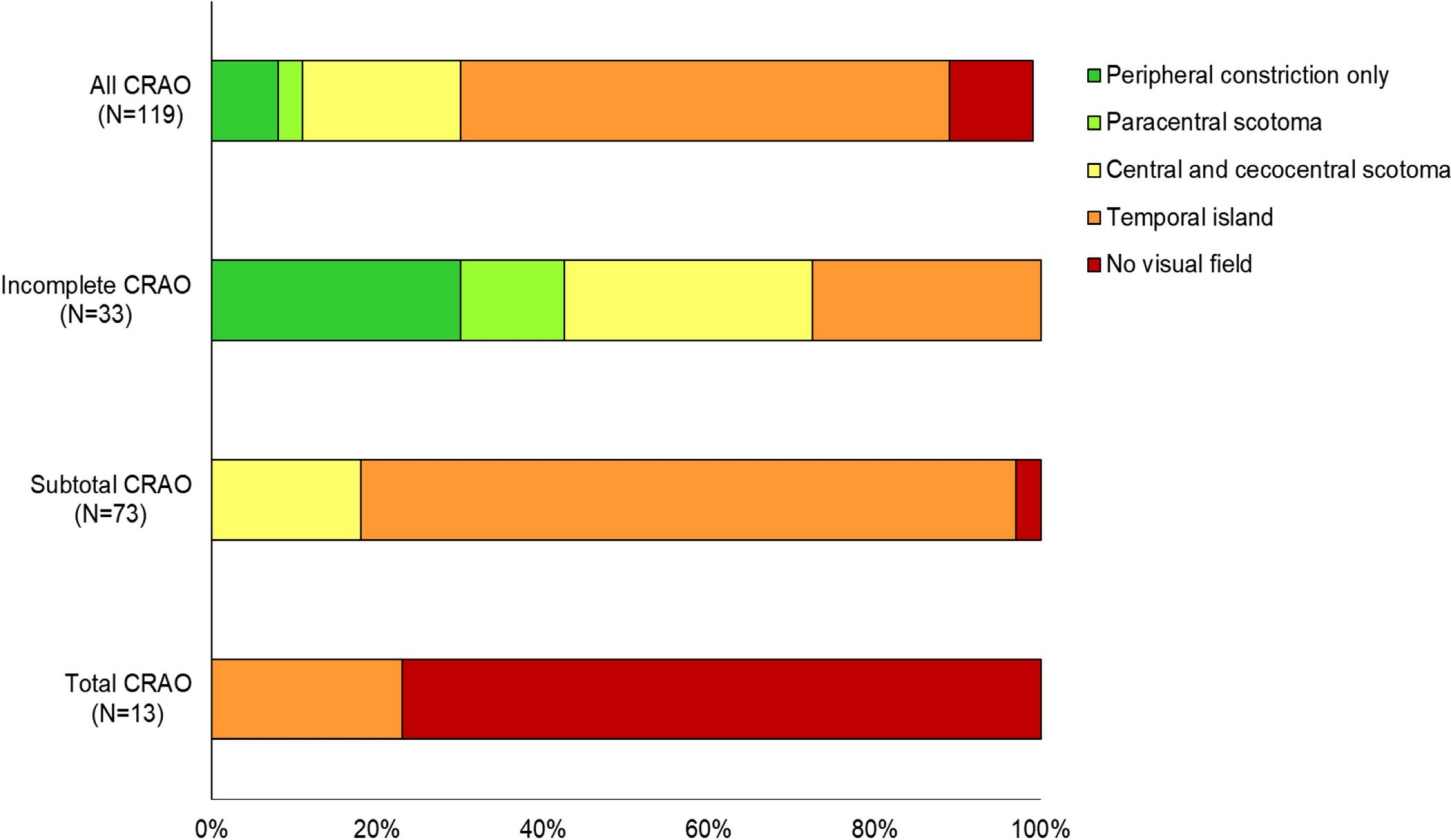
Bar graphs showing the proportion of initial visual field defect types according to the stage of CRAO. There was a significant difference in types of visual field defect among the CRAO stages (p<0.001).

We observed five characteristic types of VFDs that were associated with CRAO stages (P < 0.001, Table 1 and Fig 2): peripheral constriction only (8%), paracentral scotoma (3%), central and cecocentral scotoma (19%), temporal island (59%), and no visual field (10%). In the incomplete CRAO group, 10 (30%) patients had peripheral constriction only, 4 (13%) patients had paracentral scotoma, 10 (30%) patients had central and cecocentral scotoma, and 9 (27%) patients had temporal islands. In the subtotal CRAO group, 13 (18%) patients had central and cecocentral scotoma, 58 (80%) patients had temporal islands, and 2 (2%) patients had no visual field. In the total CRAO group, 3 (23%) patients had temporal islands and 10 (77%) patients had no visual field. We also analyzed the association between baseline parameters and severe VFDs, including “temporal island” and “no visual field.” Severe stages of CRAO, poor baseline BCVA, delayed FA arm-to-retina time, thick baseline CMT, and poor baseline OCT morphological features showed statistically significant associations with baseline severe VFDs (all *P* < .001 except *P* = .037 in FA arm-to-retina time, Table 2).

**Table 2:** Association of baseline severe VFDs (including “temporal island” and “no visual field”) with baseline clinical variables in eyes with CRAO (based on logistic regression analyses).

Variables	Baseline VFDs		Severe vs Mild VFDs	
	Severe VFDs (N = 82)	Mild VFDs (N = 37)	OR (Range with 95% C.I.)	*P*^†^
Age	61.11±16.22	60.27±13.0		.782
Gender				.596
Male	49 / 73 (67%)	24 / 73 (33%)	1.24 (0.56–2.79)	
Female	33 / 46 (72%)	13 / 46 (28%)	0.80 (0.36–1.80)	
Mean time from symptom onset to treatment (hour)	24.70±35.94	48.73±70.78		.060
Stage of CRAO				**< .001**
Incomplete CRAO	9 / 33 (27%)	24 / 33 (73%)	0.07 (0.03–0.18)	
Complete CRAO	73 / 86 (85%)	13 / 86 (15%)	14.97 (5.69–39.38)	
Subtotal CRAO : Total CRAO	60 : 13	13 : 0		
Baseline best-corrected visual acuity (logMAR)	2.36±0.33	1.92±0.50		**< .001**
FA arm-to-retina time (sec)^‡^	24.83±9.45	21.14±7.07		**.037**
Baseline central macular thickness (um)^‡^	431.76±174.50	302.81±87.98		**< .001**
Baseline OCT morphologic features^‡^				**< .001**
Inner retinal hyperreflectivity only	16 / 45 (36%)	29 / 45 (64%)	0.07 (0.03–0.18)	
Retinal thickening and loss of layer structure	49 / 55 (89%)	6 / 55 (11%)	8.44 (3.15–22.60)	
Retinal fluid	14 / 16 (88%)	2 / 16 (12%)	3.77 (0.81–17.54)	

*P* values in boldface indicate statistical significance.

† Mann-Whitney U test for baseline best-corrected visual acuity, and baseline central macular thickness; independent T-Test for age and mean time from symptom onset to treatment, FA arm-to-retina time; chi-square test for gender, stages of CRAO, baseline OCT morphologic features

‡ Data analyzed for all patients, except for one patient who did not undergo optical coherence tomography and fluorescein angiography.

C.I., confidence interval; CRAO, central retinal artery occlusion; FA, fluorescein angiography; logMAR, logarithm of the minimal angle of resolution; OCT, optical coherence tomography; OR, odds ratio; VFDs, visual field defects

**Fig 3:**
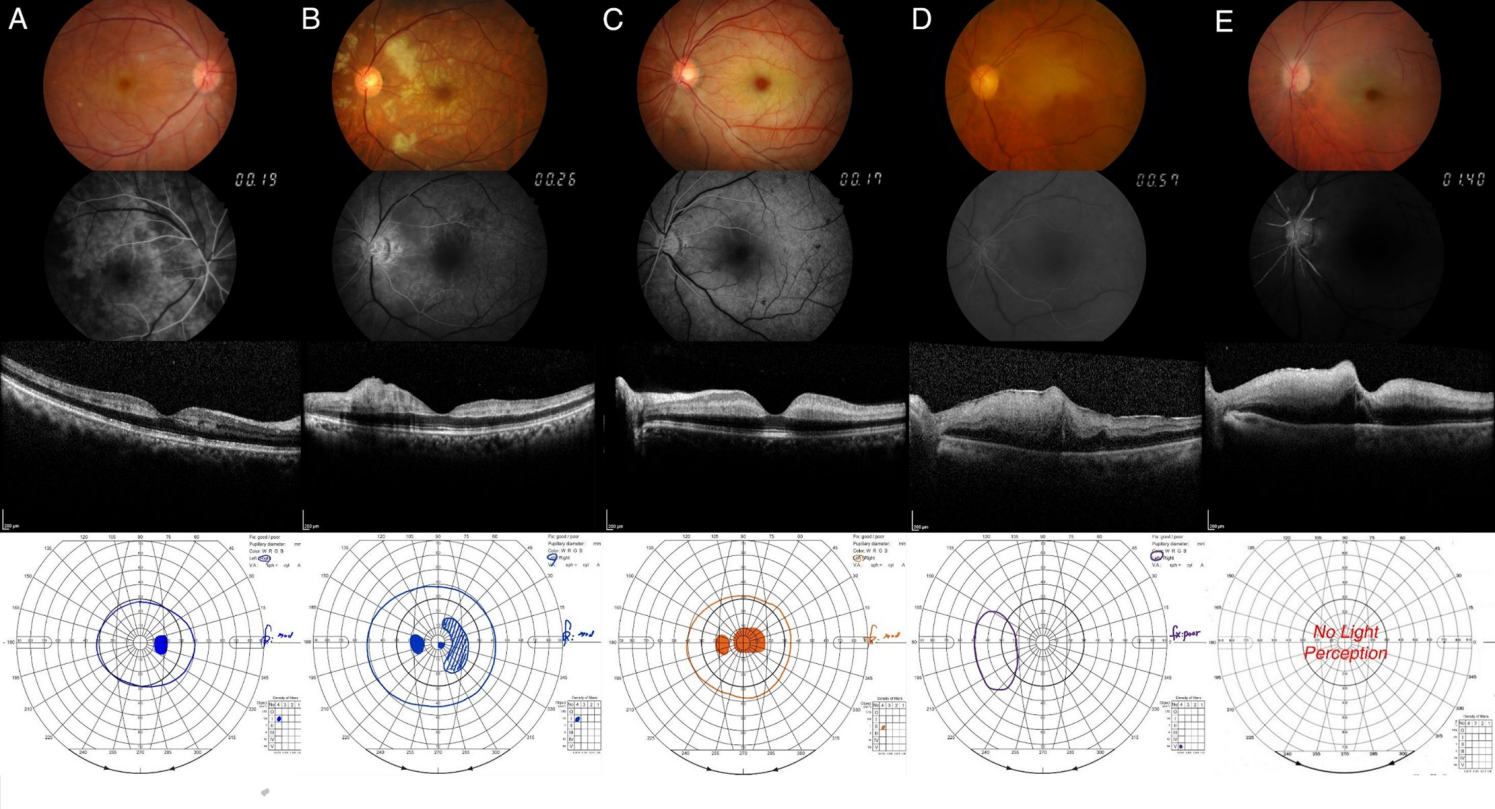
Eyes with central retinal arterial occlusion (CRAO) showing 5 characteristic visual field types. (From left to right) Cases with 5 characteristic visual fields: peripheral constriction, paracentral scotoma, central scotoma, temporal island, no light perception. (From top to bottom) fundus photography, fundus fluorescein angiography, spectral-domain optical coherence tomography, and Goldmann perimetry of the eyes with CRAO.

**Fig 4:**
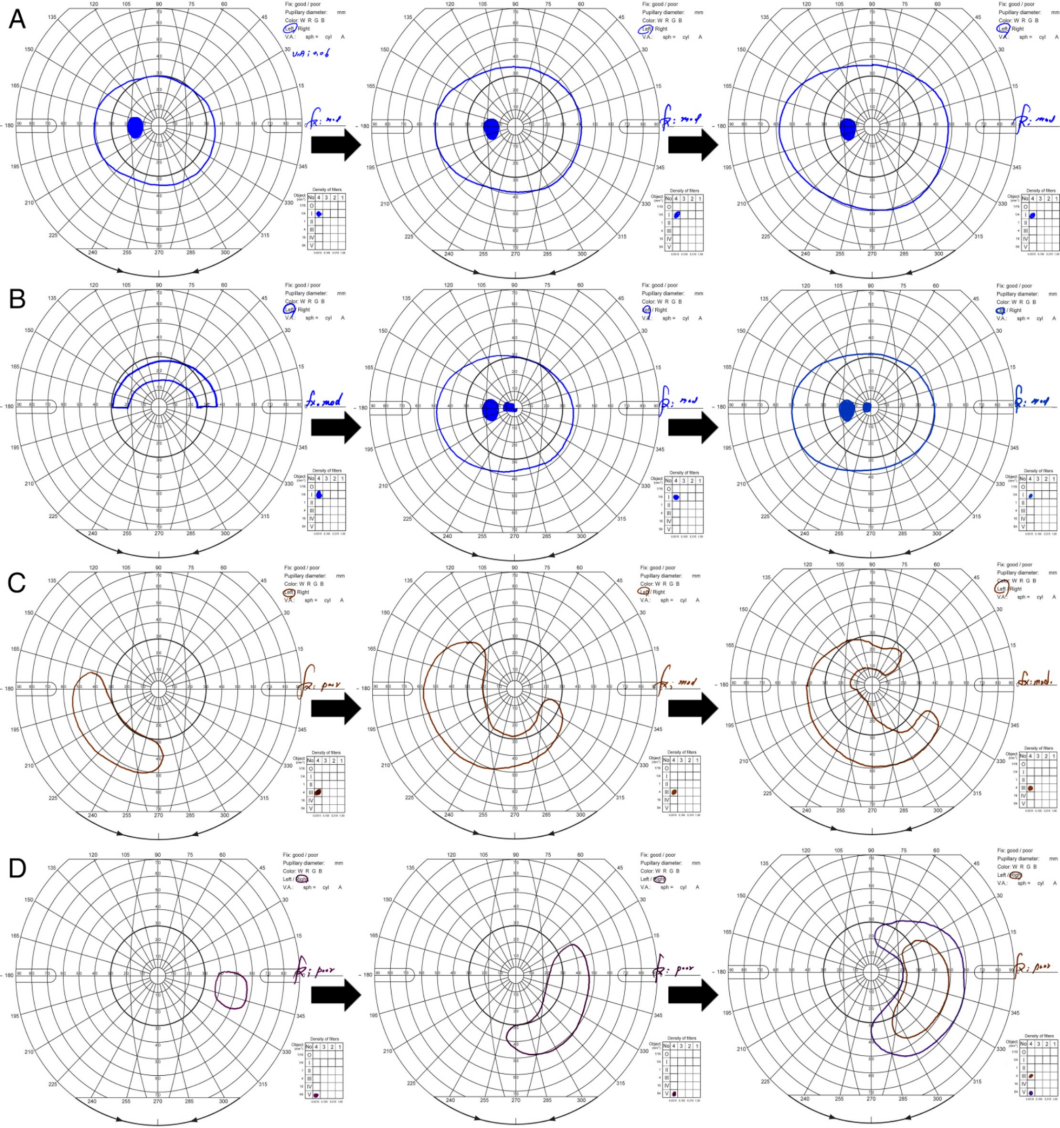
Improvement of visual field defect overtime in eyes with central retinal arterial occlusion. (From left to right) initial, 1 month, and 6 months after the symptom onset.

We observed visual field improvement in 28 of 72 patients with CRAO (39%). The incidence of visual field improvement had a significant association with the CRAO stages (*P* < 0.001, Table 1), implying that the more severe the CRAO stage, the lower the chance of improvement in the visual field: incomplete, 64%; subtotal, 24%; and total, 0%. Representative images of the fundus photography, fluorescein angiography, SD-OCT, Goldmann perimetry for each type of visual field defect are shown in Fig 3. Additionally, several cases of visual field improvements are shown in Fig 4. The remnant peripheral and paracentral fields gradually recovered while the central field was the last to recover. We analyzed the association between baseline parameters, treatment, and visual field improvement (Table 3). Among the clinical factors examined, mild stages of CRAO, good baseline OCT morphological features, and mild baseline VFDs showed a statistically significant association with visual field improvement (*P* < .001, *P* = .012, and *P* < .001, respectively). The mean time from symptom onset to treatment showed no statistical significance in univariate analysis (*P* = .298) and multivariate analysis (*P* = .802). Thus, the time factor should not be considered as the confounding factor in the multivariate analysis model, summarized in Table 3.

**Table 3:** Association of visual field improvement with clinical variables in eyes with CRAO.

Variables	Visual field improvement* (N = 28)	No improvement* (N = 44)	Univariate analysis†		Multivariate analysis (Model I)^§^		Multivariate analysis (Model II)^§^	
			OR (Range with 95% C.I.)	*P*	OR (Range with 95% C.I.)	*P*	OR (Range with 95% C.I.)	*P*
Age	58.9±14.1	59.6±13.8		.873				
Gender				.576				
Male	19 / 46 (41%)	27 / 46 (59%)	1.33 (0.49–3.61)					
Female	9 / 26 (35%)	17 / 26 (65%)	0.75 (0.28–2.04)					
Mean time from symptom onset to treatment (hour)	43.3±51.2	28.9±58.3		.298				
Stage of CRAO				**< .001**				
Incomplete CRAO	18 / 28 (64%)	10 / 28 (36%)	6.12 (2.15–17.42)		1			
Complete CRAO	10 / 44 (23%)	34 / 44 (77%)	0.163 (0.06–0.47)		0.14 (0.05–0.43)	**.001**		
Subtotal CRAO : Total CRAO	10 : 0	31 : 3						
Baseline best-corrected visual acuity (logMAR)	1.94±0.53	2.25±0.43		**.004**				
FA arm-to-retina time (sec)^‡^	22.04±7.95	24.33±8.74		.268				
Baseline central macular thickness (um)^‡^	327.50±110.84	402.02±169.46		.063				
Baseline OCT morphologic features^‡^				**.012**				
Inner retinal hyperreflectivity only	19 / 33 (58%)	14 / 33 (42%)	4.37 (1.58–12.1)					
Retinal thickening and loss of layer structure	8 / 31 (26%)	23 / 31 (74%)	0.35 (0.13–0.96)					
Retinal fluid	1 / 7 (14%)	6 / 7 (86%)	0.23 (0.03–2.01)					
Severity of baseline visual field defect^¶^				**< .001**				
Mild visual field defect	21 / 29 (72%)	8 / 29 (28%)	13.5 (4.28–42.56)				1	
Severe visual field defect	7 / 43 (16%)	36 / 43 (84%)	0.07 (0.02–0.23)				0.06 (0.02–0.21)	**< .001**
Treatment				.816				
Intra-arterial thrombolysis	19 / 50 (38%)	31 / 50 (62%)	0.89 (0.32–2.47)		1.67 (0.50–5.61)	.409	2.32 (0.59–9.23)	.231
Conservative treatment	9 / 22 (41%)	13 / 22 (59%)	1.13 (0.41–3.15)		1		1	

*P* values in boldface indicate statistical significance.

* Visual field improvement was evaluated in only those who underwent at least two sequential visual field tests (N = 72)

† Mann-Whitney U test for age, baseline best-corrected visual acuity, and baseline central macular thickness; independent T-Test for mean time from symptom onset to treatment, FA arm-to-retina time; fisher’s exact test for baseline OCT morphologic features; chi-square test for gender, stages of CRAO, intraarterial thrombolysis

‡ Data analyzed for all patients, except for one patient who did not undergo optical coherence tomography and fluorescein angiography.

§ Stepwise multiple logistic regression analyses. Stage of CRAO and treatment were entered as variables in Model I; severity of initial visual field defect and treatment were entered as variables in Model II Two separate analyses were done due to the multicollinearity between two variables, stage of CRAO and severity of initial visual field defect.

¶ Mild visual field defect includes peripheral constriction, paracentral scotoma, central and cecocentral scotoma, and severe visual field defect includes temporal island and no visual field.

C.I., confidence interval; CRAO, central retinal artery occlusion; FA, fluorescein angiography; logMAR, logarithm of the minimal angle of resolution; OCT, optical coherence tomography; OR, odds ratio; VFDs, visual field defects

Among the 50 patients with IAT performed, 19 (38%) patients showed visual field improvement. Compared with the conservative treatment, the rate of visual field improvement in IAT group was not significantly different (*P* = .816, Table 3). The odds ratio for visual field improvement was 1.67 (0.50–5.61) after adjustment for CRAO stages and 2.32 (0.59–9.23) after adjustment for baseline types of visual field defect, favoring IAT without statistical significance.

## Discussion

The mechanism of the hypoxic retinal damage results in VFDs in CRAO. [16–19] The retina is a metabolically active tissue, and acute hypoxia by the occlusion of supplying arteries causes retinal ischemia, which presents as the opacification and edema of the retinal nerve fiber and ganglion cell layer. Moreover, severe retinal ischemia could lead to outer retinal thinning and photoreceptor defects in the macula.^9^ Therefore, the retinal ischemic damage from impaired arterial perfusion contributes to the visual loss and VFDs. Retinal hypoperfusion and ischemia can vary depending on the retinal location in relation to the distance from the optic nerve head and the central macula. McLeod depicted the area of retinal hypoperfusion as the 3 oxygenation-based tissue compartments: anoxic posterior pole (infarct area), hypoxic mid-periphery named as “ischemic penumbra,” and normoxic peripheral retinal tissue. [19] Moreover, Hayreh suggested that the mechanism for the central scotoma is ischemic damage in the central macular region, while peripheral lesions are relatively maintained due to the choroidal vascular supply. [20, 21] As the density of photoreceptors is highest in the central macula, we can postulate that the retinal cells in the central macular area have the highest oxygen demand; therefore, they are the most vulnerable to ischemic injury in acute CRAO. Thus, central scotoma is frequently observed as the main visual field defect in the early stages of CRAO. However, if the cilioretinal artery is spared, the central macular area can be saved. As the retinal ischemic damage worsens, the nasal side of the optic disc becomes the last preserved area, which is supplied by the retinal and ciliary arteries, leading to the representative “temporal island” VFD.

**Fig 5:**
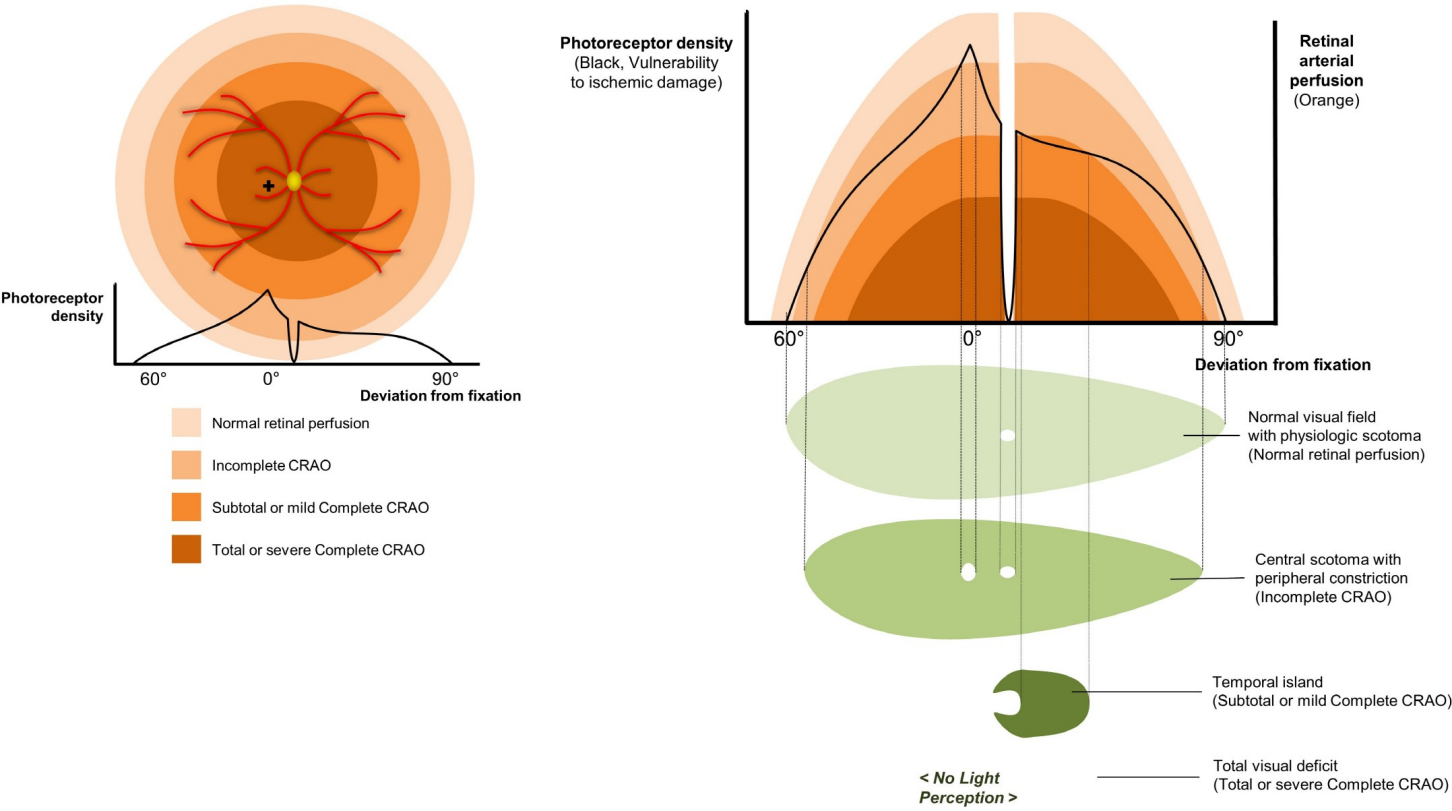
Fundus topography and related visual field defects in acute non-arteritic central retinal artery occlusion (CRAO). (Left) Schematic diagram of perfusion-based retinal zones (orange-colored) and retinal photoreceptor density (black solid line), which can be interpreted as “oxygen consumption” or “a vulnerability to ischemic damage”. ‘+’ marks fovea. (Right) Descriptive diagram of how various retinal perfusion status(orange-colored) lead to characteristic visual field defect types (green-colored). This diagram is based on the concept that the shape of the visual field defect is determined by the balance between the ischemic vulnerability and the perfusion status of each retinal region. In an eye with mildly impaired retinal arterial perfusion (Incomplete CRAO), a macula, which has the highest photoreceptor density can be damaged first, causing central scotoma. This can be accompanied by mild peripheral constriction. In an eye with more impaired retinal arterial perfusion (subtotal or mild Complete CRAO), the residual functional visual field is often confined to an island of temporal visual field. No light perception can be expected in the total or severe complete CRAO eyes.

Schematic images in Fig 5 may explain how various retinal perfusion statuses lead to characteristic visual field types. The left image describes the retinal perfusion statuses as orange-colored concentric rings, which is similar to McLeod’s descriptions. Photoreceptor density, drawn with a black solid line, is thought to be related to the metabolic needs of retinal tissue, thus, it can be interpreted as vulnerable to hypoxic tissue damage. Based on the concept that visual field is determined by the balance between the vulnerabilities of each retinal region and the perfusion status, the right image shows how the shape of functional visual fields forms. In an eye with mildly impaired tissue perfusion (Incomplete CRAO), a macula, which has the highest photoreceptor density in the whole retina, can be damaged first, accompanied by mild peripheral constriction. In an eye with more severely impaired retinal arterial perfusion (subtotal or mild complete CRAO), the residual visual field is often confined to an island of temporal visual field. No light perception can be expected in total or severe complete CRAO eyes where ciliary arterial perfusion is often diminished.

Hayreh and Zimmerman described the frequencies of the types of VFDs in both central 30-degree and peripheral field in patients with CRAO. [6] Transient or cilioretinal artery sparing non-arteritic CRAO showed relatively spared central visual fields compared to typical non-arteritic CRAO, while central scotoma was the most common type of visual field defect in CRAO. As in general visual fields, no peripheral defects or constricted field defects were common in the transient or cilioretinal artery-sparing group, while temporal islands were relatively common in the typical non-arteritic group. There were large percentages of visual field improvement (normal, better, or stable) in the central and peripheral visual fields, while only less than 15% showed visual field worsening. In our study, visual field tests showed improvement in 39% of patients, though it cannot be compared because criteria defining ‘visual field improvement’ differ between the two studies.

In our previous study, we revealed that structural changes including the initial inner and outer retinal thickening, baseline macula edema, final retinal thinning, and central macular thickness at the initial and final presentation differed significantly among CRAO stages. [9] In this study, we reconfirmed that the retinal morphologic abnormalities are significantly associated with the CRAO stage. In addition, baseline OCT structural changes and central macular thickness are correlated with the types of VFDs.

We also investigated the association between the IAT treatment and visual field improvement. In our previous report, the IAT group exhibited visual improvement in terms of visual acuity in eyes with incomplete CRAO, but not in the subtotal and total CRAO. [8] In this study, we discovered that visual field improvement has no significant association with the treatment method (IAT vs conservative treatment), while improvement in visual acuity was associated with the mild or incomplete stages of CRAO and corresponding clinical features such as mild VFDs and mild OCT findings. However, after adjusting for the stage of CRAO or baseline VFDs, the analysis indicates a preference for IAT, with an odds ratio of 1.67 or 2.32 without statistical significance. This result suggests that compared to the EAGLE trial [10] mentioned above, the opportunistic tendency toward visual field improvement in patients with IAT treatment should not be neglected, even though there were no definite statistical significance. Since the number of included subjects with potential visual field improvement is small (33 patients in the incomplete stage group), further studies with a larger number of subjects with sufficient statistical power is needed to reveal the true efficacy of IAT in terms of visual field improvement.

The present study has several limitations. First, although we enrolled a large number of subjects in the study, only 72 patients (61%) underwent follow-up examinations including Goldmann perimetry, which was not sufficient to compare the temporal changes between subgroups. The dropouts may have affected the analysis of visual field changes. Second, the retrospective nature of the study had inherent drawbacks of selection bias; in particular, the time between CRAO onset and treatment and treatment choices, which could not be controlled. Additionally, the time from the symptom onset to the initial visit varied from 1 hour to 14 days, which might influence baseline OCT and Goldmann perimetry findings. Moreover, the variability of the follow-up period for each patient might be a source of bias in the evaluation of final visual outcomes.

In conclusion, there are five characteristic types of VFDs in eyes with CRAO and the types and their improvements are associated with the severity of retinal ischemia. The balance between the retinal arterial perfusion and the retinal ischemic vulnerability might be the determining mechanism of types of VFDs. The remnant visual field in CRAO patients would be important in terms of quality of life even in patients with poor central visual acuities.

## Supporting information

S1 File Supporting dataset for CRAO patients investigated. (XLSX)
