# Correction: Visual field defects and changes in central retinal artery occlusion

**DOI:** 10.1371/journal.pone.0218363

**Published:** 2019-06-10

**Authors:** Hyeong Min Kim, Young Joo Park, Kyu Hyung Park, Se Joon Woo

## Notice of republication

An incorrect version of S1 File was published in error. This article was republished on June 3, 2019 to correct for this error. Please download this article again to view the correct version.
